# Supplementary material for: Acceleration of dolomitization by zinc in saline waters
Source: Nat Commun. 2019 Apr 23;10:1851. doi: 10.1038/s41467-019-09870-y (PMC6478858; doi:10.1038/s41467-019-09870-y)
Supplement: Supplementary file 1 — Supplementary Information [file 41467_2019_9870_MOESM1_ESM.pdf]

Supplementary Information

Acceleration of Dolomitization by Zinc in Saline Waters

Vandeginste et al.

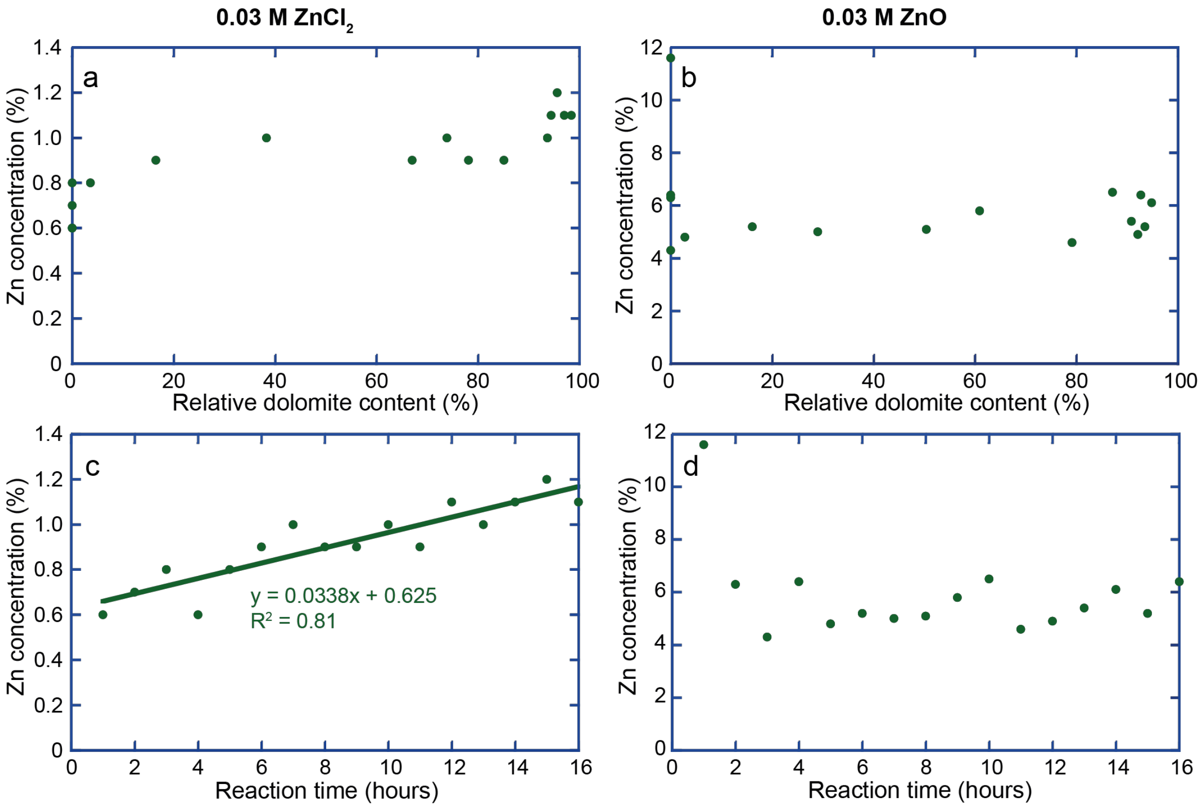

Supplementary Figure 1. Plots of zinc concentration in reaction products against relative dolomite content (related to dolomitization reaction progress) and reaction time

The concentration of Zn in the reaction products from both, 0.03M $ZnCl_2$ (a) and 0.03M ZnO (b) experiments show that there is no clear correlation between the Zn content and the percentage of dolomite in the precipitates, suggesting Zn is not incorporated in the dolomite structure, but rather present as simonkolleite and zincite, as confirmed by PXRD data. The Zn concentration in the reaction products from the 0.03M $ZnCl_2$ experiment increases as a function of reaction time (c). In contrast, the Zn concentration decreases quickly and then stabilizes in the 0.03M ZnO experiment (d), indicating the dissolution of zincite in the first three hours and the formation of simonkolleite later, consistent with PXRD data.

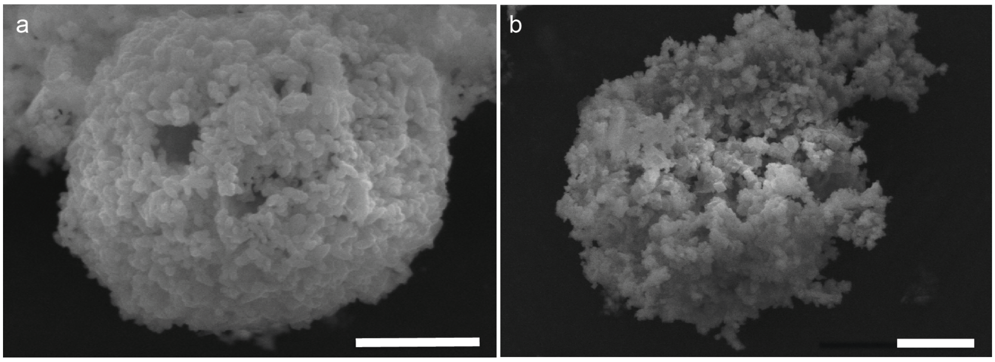

Supplementary Figure 2. SEM images of reaction products

(a) Product after 8 hours of reaction in the Zn-free saline solution at 200ºC. The solid material contains calcite only. (b) Product after 14 hours of reaction in the $ZnCl_2$ saline solution at 200ºC. The precipitate consists completely of ordered crystalline dolomite. The scale bar represents 5 μm (a, b).

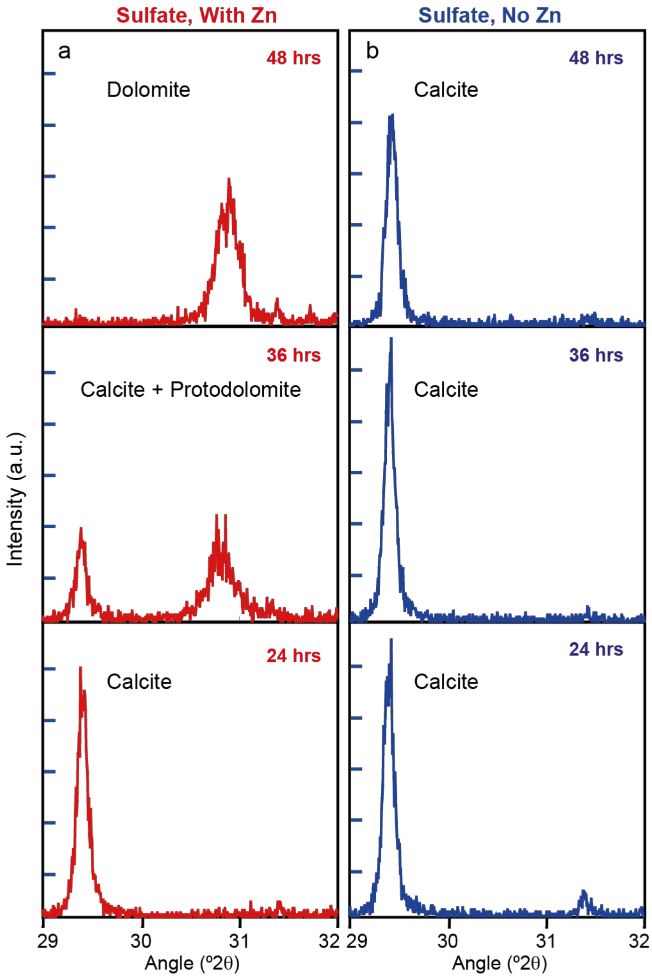

Supplementary Figure 3. Powder X-ray diffractograms (selected 29-32°2θ) of reaction products at selected time intervals of 24, 36 and 48 hours

These data demonstrate the significant impact of 0.2 weight% Zn (a) on the dolomitization rate in sulfate-containing saline fluids at 200 °C (b). The reaction products contain besides carbonate also anhydrite, which forms readily by the presence of calcium and sulfate in the solutions.

$ZnCl_2$ concentration (M)	(Proto)dolomite (%)		
	Average	Number of analyses	Stdev
0.01	11	2	0
0.03	61	5	4
0.05	77	3	2
0.1	86	2	3
0.2	99	2	1

Supplementary Table 1 (Proto)dolomite percentage as a function of Zn concentration in the $ZnCl_2$ experiments at 200 ºC with 8 hours reaction time (The percentage is derived from PXRD analyses of the reaction products)

Time (hours)	(Proto)dolomite (%)		
	Average	Number of analyses	Stdev
4	0	1	
8	0	1	
12	3	4	2
13	7	3	4
14	15	3	2
15	28	3	6
16	49	4	3
17	44	3	1
18	54	3	2
19	98	3	3
20	91	4	7
21	80	3	2
22	92	3	3
23	99	3	1
24	100	4	0
25	100	3	0
26	100	3	0
27	100	3	0
32	100	2	0
36	100	1	
40	100	1	
44	100	1	
48	100	1	
52	100	1	
56	100	1	

Supplementary Table 2 (Proto)dolomite percentage as a function of reaction time in the control series experiments at 200 ºC (Percentage is derived from PXRD analyses of the reaction products)

Time (hours)	(Proto)dolomite (%)		
	Average	Number of analyses	Stdev
1	0	1	
2	0	1	
3	0	1	
4	0	3	0
5	5	3	2
6	16	3	4
7	42	3	5
8	61	5	4
9	69	3	9
10	79	3	5
11	90	3	6
12	97	3	3
13	97	3	2
14	97	3	2
15	97	3	1
16	97	3	2
20	96	1	
24	97	1	

Supplementary Table 3 (Proto)dolomite percentage as a function of reaction time in the 0.03M $ZnCl_2$ experiments at 200 ºC (Percentage is derived from PXRD analyses of the reaction products)

**Time (hours)**	**Ca (%)**	**Mg (%)**	**Zn (%)**
1	36.0	0.3	0.6
2	35.1	0.6	0.7
3	32.1	0.9	0.8
4	32.5	0.9	0.6
5	31.9	1.4	0.8
6	30.7	3.3	0.9
7	30.3	5.3	1.0
8	23.7	7.8	0.9
9	22.2	8.6	1.0
10	23.1	7.8	1.0
11	23.5	9.7	0.9
12	20.6	11.0	1.1
13	21.4	10.5	1.0
14	19.8	11.4	1.1
15	21.6	12.0	1.2
16	20.3	10.8	1.1

Supplementary Table 4 Contents of Ca, Mg and Zn as a function of reaction time in the 0.03M $ZnCl_2$ experiments at 200 ºC (Geochemical data are based on ICP-OES measurements of the reaction products)

**Time (hours)**	**Calcite (%)**	**(Proto)dolomite (%)**	**Simonkolleite (%)**
1	100	0	0
2	100	0	0
3	100	0	0
4	100	0	0
5	96	4	0
6	84	17	0
7	62	38	0
8	33	67	0
9	20	78	2
10	26	74	0
11	12	85	3
12	0	98	2
13	4	94	3
14	0	94	6
15	0	96	4
16	1	97	2

Supplementary Table 5 Relative mineral abundance as a function of reaction time in the 0.03M $ZnCl_2$ experiments at 200ºC (Relative mineral percentages are derived from PXRD analyses of the reaction products)

Time (hours)	(Proto)dolomite (%)		
	Average	Number of analyses	Stdev
1	0	1	
2	0	2	0
3	0	1	
4	0	2	0
5	3	1	
6	14	2	3
7	29	1	
8	55	2	6
9	61	1	
10	82	2	8
11	79	1	
12	92	2	0.4
13	91	1	
14	95	1	
15	94	1	
16	93	1	
17	95	1	
18	93	1	
19	91	1	
20	94	1	
21	93	1	
22	94	1	
23	93	1	
24	92	1	
28	93	1	

Supplementary Table 6 (Proto)dolomite percentage as a function of reaction time in the 0.03M ZnO experiments at 200 ºC (Percentage is derived from PXRD analyses of the reaction products)

Time (hours)	Ca (%)	Mg (%)	Zn (%)
1	35.5	1.2	11.6
2	30.3	2.5	6.3
3	28.2	3.1	4.3
4	31.7	4.0	6.4
5	31.8	4.2	4.8
6	28.4	5.8	5.2
7	27.9	7.2	5.0
8	22.9	9.2	5.1
9	24.7	10.4	5.8
10	19.6	12.7	6.5
11	22.2	11.1	4.6
12	18.9	13.1	4.9
13	18.9	12.5	5.4
14	18.4	13.9	6.1
15	19.4	12.7	5.2
16	19.9	13.4	6.4

Supplementary Table 7 Contents of Ca, Mg and Zn as a function of reaction time in the 0.03M ZnO experiments at 200 ºC (Geochemical data are based on ICP-OES measurements of the reaction products)

Time (hours)	Calcite (%)	Zincite (%)	(Proto)dolomite (%)	Simonkolleite (%)
1	76	24	0	0
2	86	11	0	3
3	96	0	0	4
4	95	0	0	5
5	92	0	3	5
6	78	0	16	6
7	66	0	29	5
8	43	0	50	6
9	31	0	61	8
10	3	0	87	10
11	14	0	79	7
12	1	0	92	7
13	2	0	91	7
14	0	0	95	5
15	0	0	94	7
16	0	0	93	7

Supplementary Table 8 Relative mineral abundance as a function of reaction time in the 0.03M ZnO experiments at 200ºC (Relative mineral percentages are derived from PXRD analyses of the reaction products)

Time (hours)	Protodolomite* (%)
12	0
18	0
24	0
30	0
36	0
48	0
60	0
72	16

Supplementary Table 9 Percentage of protodolomite to total carbonate ratio (*thus not taking account of the anhydrite present in the reaction product) as a function of reaction time in the 0.03M $MgSO_4$ experiment at 200 ºC (Mineral contents in the reaction products are derived from PXRD analyses)

Time (hours)	(Proto)dolomite* (%)
12	0
18	0
24	0
30	1
36	65
48	100
60	100
72	100

Supplementary Table 10 Percentage of (proto)dolomite / (total carbonate) ratio (*thus not taking account of the anhydrite present in the reaction product) as a function of reaction time in the 0.03M $ZnSO_4$ experiment at 200 ºC (Mineral contents in the reaction products are derived from PXRD analyses)
